# Influence of Polypropylene and Steel Fibers on the Performance and Crack Repair of Self-Compacting Concrete

**DOI:** 10.3390/ma14195506

**Published:** 2021-09-24

**Authors:** Mohammed A. Abed, Jan Fořt, Abdulkarim Naoulo, Amr Essa

**Affiliations:** 1 Department of Civil and Environmental Engineering, Rutgers, The State University of New Jersey, Piscataway, NJ 08854, USA; mohammed.abed@rutgers.edu; 2 Department of Materials Engineering and Chemistry, Faculty of Civil Engineering, Czech Technical University in Prague, 16629 Prague, Czech Republic; 3 Department of Structural Engineering, Budapest University of Technology and Economics, 1111 Budapest, Hungary; abdulkarim-naoulo@live.com (A.N.); amr9993essa@gmail.com (A.E.)

**Keywords:** epoxy injection, polypropylene fiber, steel fiber, mechanical properties, crack repair, workability

## Abstract

The research reported in this paper aims to evaluate the epoxy injection technique used to strengthen fiber-reinforced self-compacting concrete (FRSCC) with high strength. This method is carried out on ruptured concrete specimens to assess the efficiency of the epoxy resin adhesive injection retrofitting technique for strength and stiffness. Five FRSCC mixes were designed and placed using different types (steel and polypropylene) and contents (0%, 0.25%, and 0.45% by volume) of fibers. The fresh and mechanical properties in addition to the microstructure of produced mixes were evaluated to assess the impact of fibers on the behavior of FRSCC. Results showed that the workability of FRSCC is reduced by increasing steel or polypropylene fiber content; however, the rheological characteristics of placed mixes satisfied the European Guidelines for Self-Compacting Concrete recommendation for fresh concrete. Also, splitting tensile, flexural, and shear strengths were enhanced by increasing fiber content. The simultaneous application of epoxy injection in FRSCC for repairing damaged concrete beams was shown to be highly effective.

## 1. Introduction

The versatility and efficiency of concrete as a construction material have demonstrated its ability to become the ultimate building material in the world. Despite concrete’s compression capacity, the low tensile strength and brittle characteristics of concrete have necessitated overcoming this weakness by using several types of reinforcement (steel, fiber-reinforced polymer, fibers, etc.). Consequently, enhanced concrete service life and durability can be attained by overcoming cracks issues [1]. The influence of fibers on the workability of self-compacting concrete (SCC) requires attention during the placement of structural concrete elements. Research has proven that using randomly distributed fibers can alter the fresh and mechanical properties of concrete [2,3]. Depending on the shape and type of fibers, overcoming cracks and brittleness can be successfully achieved in cement-based materials. For the past few decades, extensive experimental work has been conducted on a wide variety of fibers including natural, synthetic, and metallic fibers, for which recommendations and guidelines have been proposed for engineering applications.

The utilization of steel fibers in concrete has been highly investigated in the past two decades, and the experimental results have proved its capacity for structural concrete applications. da Silva et al. [4] pointed out that the use of steel fibers enhances the flexural toughness and flexural strength of SCC. Other studies on the addition of steel fibers into concrete confirmed that the splitting tensile strength and modulus of rupture are enhanced as well [5]. Design codes including ACI 318 [6] and fib [7] have endorsed fibers to be used for shear reinforcement. To evaluate the shear strength of fiber-reinforced concrete elements, two different formulations are provided by the Model code. Similarly, the American code permits 0.75% as a minimum shear reinforcement of steel fibers to be used in volume fractions in normal-strength concrete beams. However, Khalo et al. [8] highlighted the negative impact of using steel fibers on the slump-flow and V-funnel times of produced (SCC) concerning volumetric content. Likewise, the corresponding compressive strength of produced SCC has decreased with attribution to workability [9]. Other issues have been reported on the use of steel fibers in concrete, including corrosion and high volumetric density, which create limitations on its use [10,11].

Considering the disadvantages of the incorporation of steel fibers in concrete, polymer-based fibers (polypropylene fibers) are considered a good alternative. Owing to their good chemical stability and hydrophobic nature, and long-term durability, performance in harsh environments can be attained [12,13]. Additionally, the use of polypropylene fibers has proven its ability to reduce the sensitivity of concrete to cracking due to shrinkage. Sun and Xu [14] reported that the use of polypropylene fibers up to 0.15% did not affect the compressive strength results of conventional concrete. Similar results were obtained for high-strength concrete with polypropylene fibers up to 0.45% volume fraction [15]. Multiple studies have been carried out to reveal the most practical way of utilizing polypropylene fibers on the mechanical concrete properties while controlling their negative impact on concrete fresh characteristics. Fiber-reinforced self-compacting concrete (FRSCC) having 0.5% fiber volume showed an adequate filling capacity [16]. To control the drop in FRSCC workability, Khayat et al. [17] suggested maintaining a constant mortar thickness over coarse aggregate and fiber inclusions. The corresponding experimental results showed that 0.75% is the critical polypropylene volume dosage and that 0.5% is the proper dosage for steel fibers. If a higher fiber dosage is needed, a viscosity-modifying agent can be used to overcome blockage and segregation of FRSCC.

Different cementitious and polymer materials are used for crack repair in reinforced concrete structures. Epoxy injection is considered an effective repair method. Based on the ACI 546 [18] “ACI Concrete Repair Guide”, epoxy resin is considered the most commonly used repair material for cracks since it possesses very good bonding and durability characteristics. According to Issa and Debs [19], if repair is required to restore the structural integrity of a crack, it should be repaired with epoxy either by injection (epoxy injection method) or by gravity flow (gravity filling method). Cracked cubes injected with epoxy showed an increase in tensile and compressive strength across the crack (around 11% reduction in the compressive strength of epoxy injected cubes, as compared to control ones with a 40% decrease) if further cracking is not anticipated. Another study conducted by Ekenel and Myers [20] has shown the impact of environmental conditioning on crack injection using carbon fiber-reinforced polymer strengthening. Results have proven an increase in stiffness in the linear region of load-displacement curves for all reinforced concrete beams injected with epoxy (the increase was noticed to be 3.5 times than control specimens).

French et al. [21] approved the efficiency of using epoxy to restore damaged specimens (over 85 % of the energy dissipation characteristics, stiffness, and strength of the original specimens are restored). Nikoupour and Nehdi studied the behavior of reinforced concrete beams after applying a low viscosity epoxy injection for repair. The study revealed an enhancement of stiffness in the linear region of the load-displacement curves of all the repaired reinforced concrete beams [22]. Ahmad et al. [23] reported in their study that the use of the epoxy injection technique to repair cracks in simply supported beams provided a considerable enhancement in the load-carrying capacity of the beams after repair (considerable 49% increase in the load-carrying capacity). On the other hand, this approach requires further verification to validate the accuracy and effectiveness [24].

The abovementioned literature review reveals that the majority of published papers on the incorporation of fibers in concrete have been restricted to the addition of fibers only; however, limited investigations have assessed the impact of epoxy injections on the flexural strengthening of FRSCC. In this regard, FRSCC mixtures modified by steel and polypropylene fibers were designed and subjected to flexural strength tests to form cracks. Consequently, the coupled effect of fibers and epoxy injection postrepairing phase to enhance the properties of FRSCC was investigated to widen the information about this type of concrete to practically apply it in the construction industry. Obtained results reveal benefits associated with the epoxy treatment of formed cracks in terms of enhancement of mechanical strength. 

## 2. Methods

### 2.1. Materials

Cement, sand, coarse aggregate, water, superplasticizer, and fibers have been used to produce the FRSCC with high strength. Thus, a low w/c ratio (0.35) and high powder content (500 Kg/m^3^) were intended for casting SCC to ensure adequate rheological properties. Ordinary Portland Cement (OPC) CEM I 42.5 N complying with the European standard [25] was used. Table 1 shows the chemical and physical characteristics of the cement.

**Figure 1:**
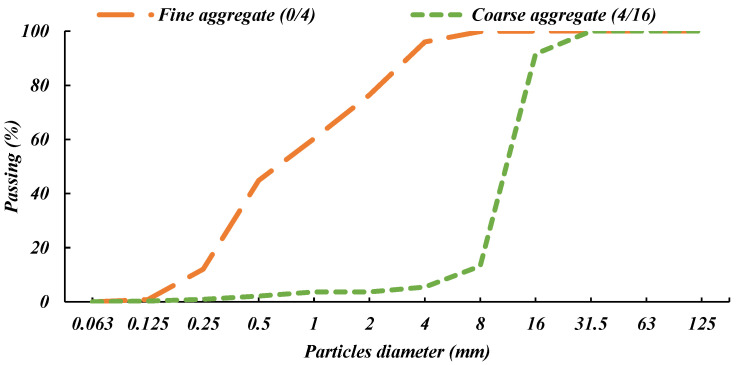
Grading curves of sand, quartz coarse aggregate, and expanded clay LWA.

Local natural quartz river (Danube river) fine and coarse aggregates were used by 45% and 55% proportions, respectively. Figure 1 shows the sieve analysis of the used aggregates. The nominal aggregate size is 16 mm; it has a serious impact on strength in the case of high-strength concrete, and it has a positive impact on fatigue performance when the fiber is used.

A modified polycarboxylates aqueous solution superplasticizer (Sika ViscoCrete-5 Neu) was used to achieve the required strength and workability under the European Guidelines for SCC [26].

It is important to note that the superplasticizer was added at the same amount for all mixes to assess the effect of steel and synthetic fibers on the fresh properties of SCC; however, this amount was optimized initially for the control mix. Two types of fibers were used in the present work: steel and synthetic fibers. The steel fiber was a 3D 65/60 BG Dramix, which is known for its advantages such as 3D, combining high performance, durability, and ease of use, and it is a time-saving and cost-efficient solution [27]. The synthetic fiber was polypropylene fiber; its type is Concrix, which is a unique bicomponent polymer fiber with a structured surface. Its high modulus of elasticity guarantees higher strength, where its special structured shell ensures excellent bonding to the concrete.

**Figure 2:**
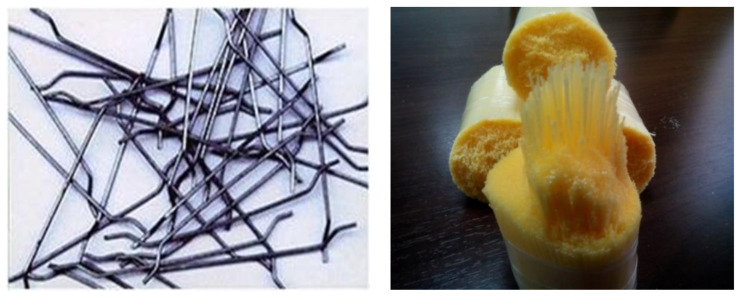
(**left**) Steel fiber and (**right**) Synthetic (polypropylene) fiber.

It is known for its advantages, such as high tensile strength and excellent postcrack behavior, in addition to its high resistance to aggressive fluids and corrosion and that provides a longer service life with minimal maintenance requirements. In addition, it can be handled easily because of its low weight. Table 2 shows the properties of both steel and synthetic fibers used, while Figure 2 presents their shape. Cracked surfaces in damaged specimens were sealed using a two-component epoxy. The specifications of the used epoxy were 30 MPa for tensile strength, 0.5% strain, 98 compressive strength MPa, and 15.9 MPa for the bond strength.

### 2.2. Mixing and Proportions

An electric mixer was used to mix the materials; it contained a flat cylindrical tank where the concrete component can be added. The tank rotated automatically for mixing, and in addition there were three steel blades that hung on the steel cover of the mixer and rotated in the opposite direction of the tank in order to make the mixing more efficient. The total mixing time was from 5.5 to 6 min, partitioned into four stages, and after each stage, the ingredients were manually mixed to achieve the highest homogeneity. In the first stage, coarse aggregates, sand, and cement were added and mixed for about 1.5 min.

In the second stage, fibers were added and mixed for 1 min, then in the third stage, the water was added to hold the components together and mixed for about 1.5 min, followed by the superplasticizer, and then mixed for 1.5 min. This procedure was optimized by the authors before starting the experimental program, and it helped to achieve the required workability and homogeneity of the SCC mix [28]. Five mixes were produced: control, which is the plain SCC, and the four mixes with different proportions of steel or polypropylene fibers (0.25 and 0.45% by volume). The fiber content was specified based on the literature and what is used locally; it was 0, 2, and 3.5 kg/m^3^ of polypropylene and 0, 20, and 35 kg/m^3^ of steel fiber. Mix proportions are presented in Table 3, where the reference mix is based on an optimized SCC with high strength [29,30].

### 2.3. Testing

To ensure that the produced mixes have satisfied the European Guidelines for SCC, slump flow diameter and V-tunnel time tests have been conducted directly after mixing. The slump flow diameter test was used to assess the workability and consistency of the concrete. The V-funnel time test was carried out on fresh FRSCC to evaluate the viscosity of the fresh concrete. Both of them were conducted per the European Guidelines for SCC [26]. Specimens were cubes of size 150 × 150 × 150 mm for the compressive strength test, prisms of 70 × 70 × 250 mm for the flexural strength test, and cylinders of Ø150 × 300 mm for the splitting tensile strength and shear strength tests. All specimens were kept in their molds for 24 h at a temperature of 22 °C ± 1 °C, then they were demolded and immersed in water for 7 days curing. Compressive strength, splitting tensile strength, and three-point flexural strength tests were conducted per European standard [31,32,33].

**Figure 3:**
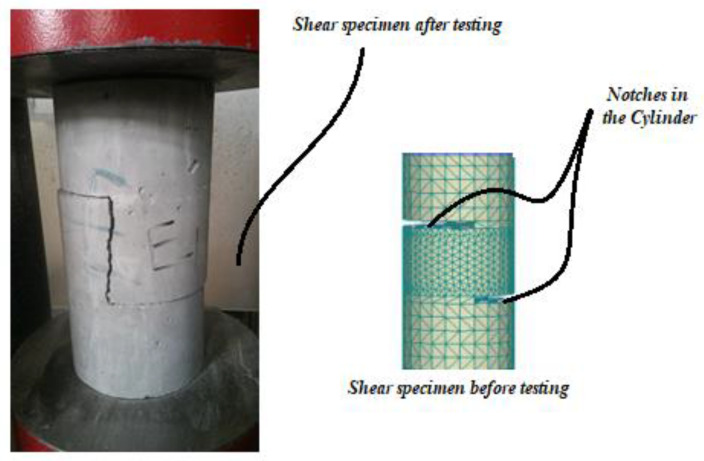
Cylindrical push-off specimen.

The shear strength was tested by the original experimental method: by using a notched cylindrical push-off specimen, which creates two stress-free zones. New boundary conditions were created to transform the compression stress into shear in a limited area. The shape of such a specimen (S-shape) allows for a longitudinal slip, which ensures the occurrence of shear stress in a plane by loading with two forces that equilibrate each other without necessitating the application of supplementary forces on the boundary to ensure equilibrium. The specimen was notched in two symmetrical areas of 1 cm thickness and 7.5 cm depth, and the spacing between the notch and base was 10 cm for both from each side; the used notched cylindrical push-off specimen is shown in Figure 3. After getting the failure load (P) for each test the compressive, splitting tensile, flexural, and shear strengths have been calculated by MPa (N/mm^2^) through the equations presented in Table 4.

**Figure 4:**
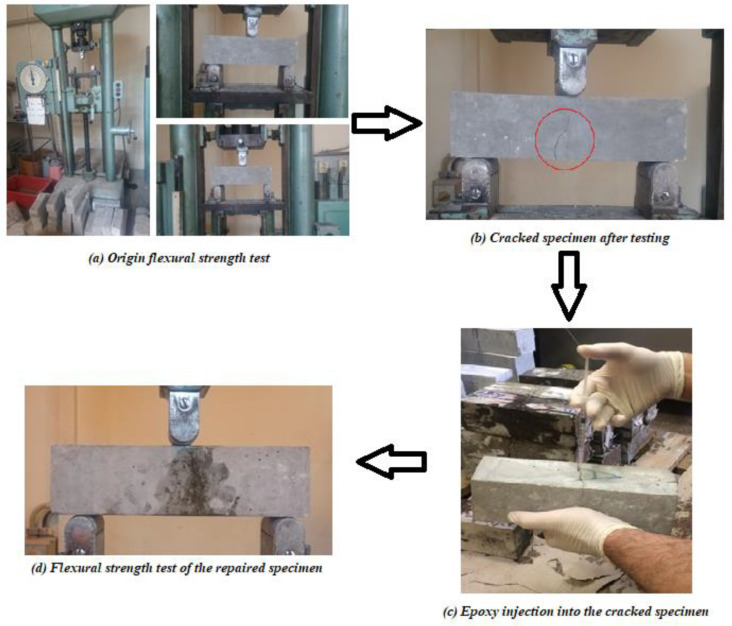
The used procedure for repairing cracked fiber concrete by epoxy injection.

By conducting the three points flexural strength test on the prism specimens, they mostly cracked on the bottom side in the middle of the span. Typically, one dominant crack appeared, as is shown in Figure 4b; however, the control specimens (without the incorporation of fiber) completely split into two halves. These cracked and split specimens were repaired using epoxy resin and tested again; the following repairing procedure was carried out on damaged specimens’ postflexural test:

1. The crack was cleaned using compressed air.
2. The epoxy resin was prepared.
3. The crack was filled with the epoxy resin by injecting it using a syringe (Figure 4c).
4. After epoxy injection, the outer edges of the crack were painted using epoxy.
5. The specimen was left for 24 h, then the flexural test was carried out on the repaired specimen (Figure 4d).

## 3. Results

### 3.1. Fresh Properties

**Figure 5:**
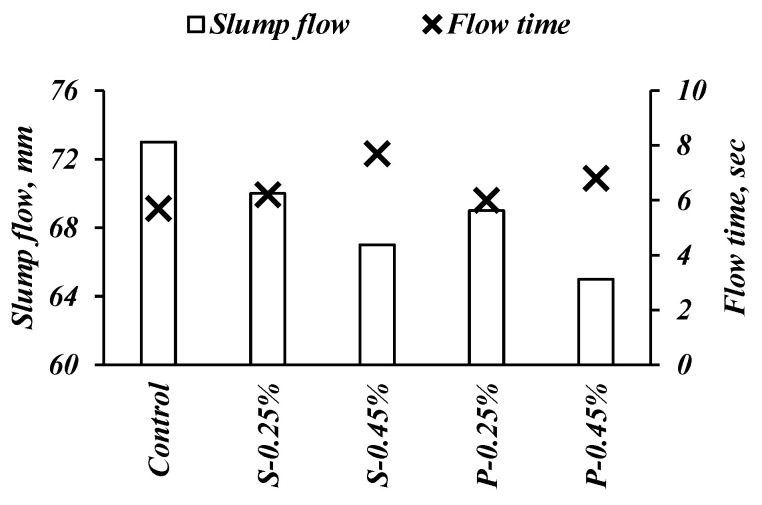
Results of slump flow diameter and V-funnel times.

The impact of steel and polypropylene fibers on FRSCC flowability and viscosity (i.e., slump flow and V-funnel time) are shown in Figure 5. Based on the European Guidelines for SCC [26], the obtained results fall within SF2 (660–750 mm) and VF1 (≤8 s) classes, reflecting adequate performance at the fresh state. Regardless of fiber type (i.e., steel or polypropylene fibers), the use of fibers led to a gradual decrease in slump flow diameter, reflecting the reduced flowability of FRSCC. For instance, slump flow spread decreased from 73 cm for the control mix without fibers to 70 and 67 cm when steel fibers increased from 0.25% to 0.45%, respectively. Similarly, the corresponding V-funnel times increased from 5.7 to 6.2 and 7.7 sec, respectively. This behavior could be attributed to the higher fiber volume that alters the flowability and viscosity of FRSCC mixes due to the increased friction with aggregates [34,35,36]. The experimental results of Yap et al. [37] reported that adding hybrid steel polypropylene fibers led to the reduced slump flow diameter of assessed FRSCC mixes, reflecting the large surface area of fibers, which necessitate more mortar inside the paste matrix.

### 3.2. Compressive Strength

**Figure 6:**
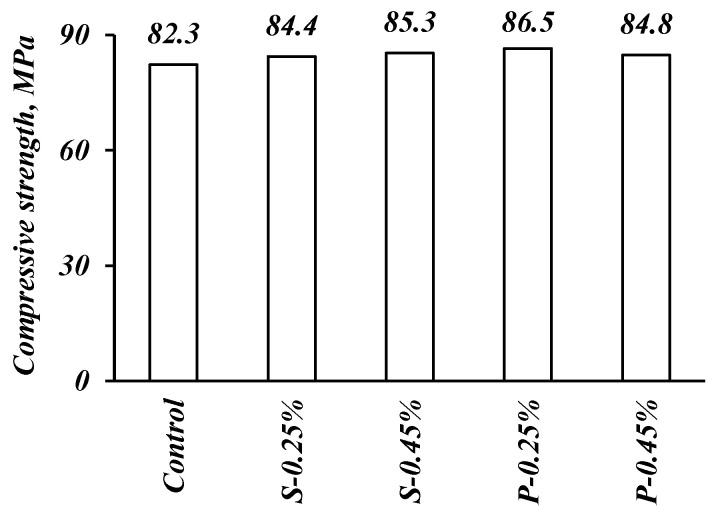
Effect of fibers on the compressive strength.

The compressive strength results of FRSCC mixes that include various volume fractions and types of fibers are illustrated in Figure 6. It can be directly noticed that the use of steel or polypropylene fibers in FRSCC does not alter the compressive strength results, since a slight increase in the compressive strength was noted (i.e., the largest increase in compressive strength was 5% for the FRSCC mix containing 0.25% of polypropylene fiber fraction) [38]. Similarly, there was a marginal increase in the compressive strength for the steel fibers mixtures with 0.25 and 0.45% fiber volume fractions in comparison to the control mix. Several studies reported the positive effect of polypropylene fibers on the compressive strength of concrete [13,39,40]. The data reported in the aforementioned studies agree with the current experimental results, implying that polypropylene fibers have limited effects on concrete compressive strength [11,41]. This enhancement may be attributed to the effect of fibers on reducing the formation and development of cracks under axial load. It is worth mentioning that the reduction of compressive strength developed by increasing the volume fraction of fibers (i.e., polypropylene fibers) can be related to the fact that increasing the volume of fibers can alter the workability of FRSCC mixes. Consequently, poorly compacted zones are formed inside the concrete, which affects the development of concrete compressive strength [42].

### 3.3. Splitting Tensile Strength

**Figure 7:**
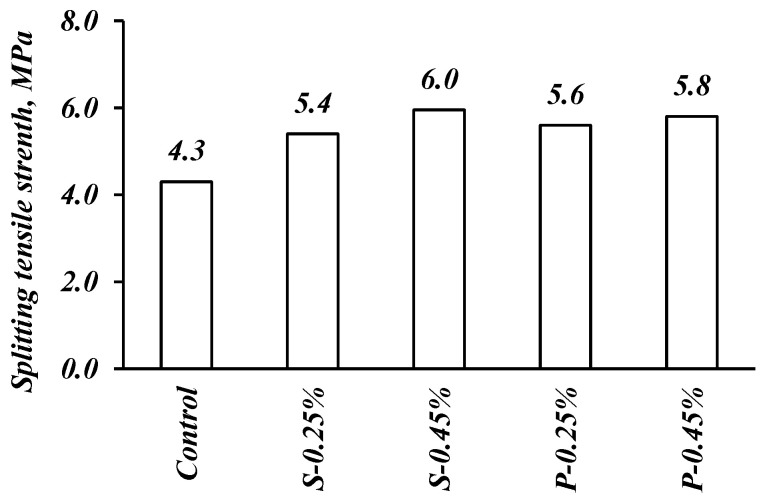
Effect of fibers on the splitting tensile strength.

To assess the impact of fibers on the tensile strength of casted FRSCC, splitting tensile strength tests were carried out on specimens, and the corresponding results are plotted in Figure 7. As expected, the addition of fibers had a significant impact on the splitting tensile strength of mixes. The positive effect of the addition of fibers is noticeable. In comparison with the control mix, the improvement in splitting tensile strength when fibers were added was in the range of 25–46%. The S-0.45% mix incorporating 0.45% by volume of steel fibers showed the highest enhancement in splitting tensile strength. Similarly, the mixes with polypropylene fibers showed enhanced performance in the splitting tensile strength, reaching up to 34% for the mix incorporating 0.45% by volume of fibers. This could be explained by the fact that fibers bridge cracks during loading, limiting their growth and propagation, thus leading to increased ultimate splitting tensile stress [9]. Results are in agreement with Yap et al. [37] and Sadrinejad et al. [43], highlighting the positive impact of polypropylene fibers in enhancing splitting tensile strength. Also, it is worth mentioning that splitting tensile strength was more vulnerable than the compressive strength results when fibers were incorporated in the FRSCC mixes at the same fiber content.

### 3.4. Shear Strength

**Figure 8:**
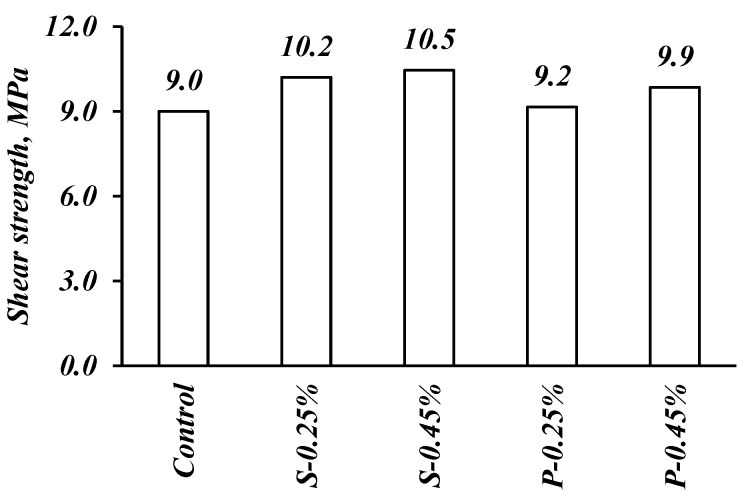
Effect of fibers on the shear strength.

According to Figure 8, the increment of shear strength is affected by fiber type and content. The use of 0.45% by volume of steel fibers in the FRSCC mix increased the shear strength by about 16% compared to that of the control mix, while using polypropylene instead of steel fibers led to a 9% increase in the shear strength. This could be related to the higher crack-arresting capacity of bridging in steel fiber with rough surfaces compared to the smooth surfaces of polypropylene fibers. The rough surface of steel fibers provides higher shear strength at the matrix/fiber interface compared to the smooth surfaces of polypropylene fibers [44]. Additionally, the use of steel fibers has proven its efficiency in enhancing the shear capacity and ductility of reinforced concrete by increasing the residual strength of concrete at the postcracking stage; fibers provide bridging stresses through the cracks so the stress is transferred across the cracked section [45,46].

### 3.5. Flexural Strength (Before and After Epoxy)

**Figure 9:**
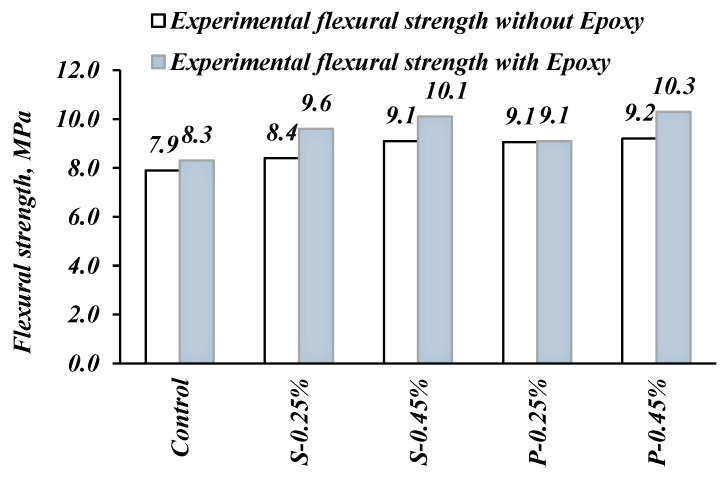
Effect of fibers on the flexural strength.

The flexural strength test results which show the effect of using steel and polypropylene fibers with and without epoxy injection are plotted in Figure 9. Regardless of the type and volume fraction of fibers, the addition of fibers in FRSCC mixes enhanced the flexural strength performance. Different results were reported in the literature on the effect of fibers on the flexural strength of cementitious mixtures. For the given fiber content, flexural strength was expected to increase. In comparison with the control mix, the improvement in flexural strength when fibers were added was in the range of 6–21%. The best performance in flexural strength corresponded to the S-0.45% mix with steel fibers. The results of the polypropylene fibers concrete point to the superior efficiency of steel fibers over polypropylene fibers, since polypropylene fibers have lower tensile strength and elastic modulus than those of steel fibers. The general improvement in the flexural strength capacity of FRSCC mixes with fibers can be attributed to the fiber capacity to limit crack propagation, which directly decreases the generation of new cracks [44]. The results of this paper agreed with Šušteršič et al. [47], who found steel fiber is so effective that it not only increases the bearing capacity of concrete after repairing the cracks with epoxy, but also enhances the concrete behavior after the first crack improves.

**Figure 10:**
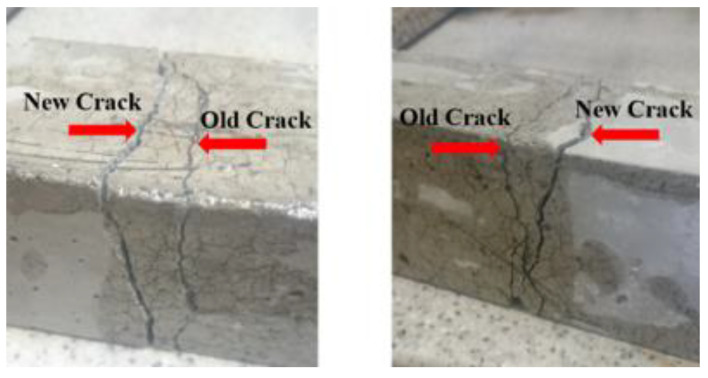
New and old cracks in the repaired reference specimens.

Based on experimental observations, all tested specimens failed within the middle third section. After breakage, beams repaired with epoxy injection performed differently. Most of the repaired control specimens that split at new different places had higher flexural strength values compared to the specimens that split at the same places (see Figure 10). In comparison with the control mix, the improvement in flexural strength when fibers were introduced was in the range of 9–24%. The best performance in flexural strength corresponded to the S-0.45% mix with steel fibers, reaching an ultimate value of 10.3 MPa. The injected epoxy is characterized by having a higher tensile strength and better strain than concrete. Therefore, as can be observed from Figure 10, the failure of concrete specimens in flexure did not occur in the region where the epoxy has been injected. The epoxy injection method provided an increase in stiffness of the tested element.

**Figure 11:**
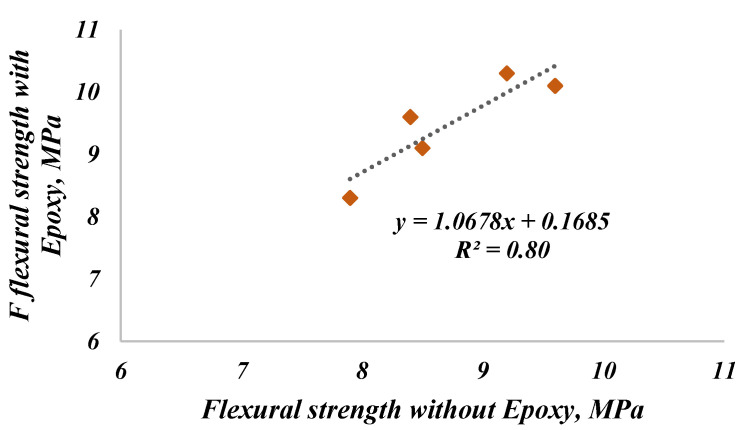
Correlation between the flexural strength of FRSCC before and after repairing with Epoxy.

The correlation between the flexural strength of the FRSCC before and after repairing by epoxy is presented in Figure 11; the correlation is linear and with a coefficient of determination of 80%. It could be concluded that repairing FRSCC is not only an economical or sustainable choice, but also a valuable one for enhancing the concrete properties. Through the presented correlation, the flexural strength of FRSCC after repairing with epoxy can be predicted. It is important to mention that this behavior was observed for the FRSCC with high strength, where its compression strength at 28 days exceeded 82 MPa for all mixtures.

**Figure 12:**
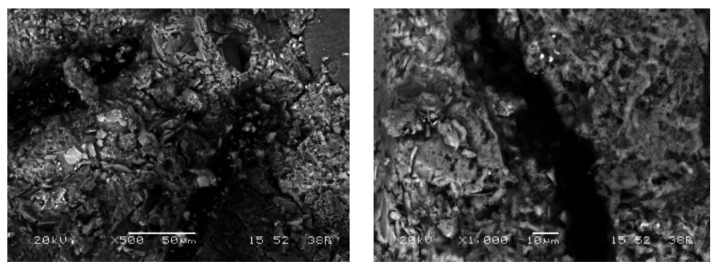
Microstructure SEM images of fiber reinforced concrete.

It has been recognized by Feng et al. [48] that the polypropylene fiber encompassed by C-S-H gel ruptured due to the low tensile strength, while steel fiber had a smooth surface that detached from its surrounding matrix. The few hydration products on the steel fiber surface imply the relatively weaker interfacial bond between steel fiber and matrix. As an attempt to explain the behavior of FRSCC after being repaired with epoxy, scanning electron microscope (SEM) images were produced to investigate the microstructure behavior of fiber-reinforced concrete, as shown in Figure 12. It can be seen that the transition zone between the fiber itself and concrete has high porosity, and one reason for the increasing flexural strength after repairing by epoxy could be this porosity. The injection of epoxy into a concrete crack introduces the epoxy resin to the pore zone between the concrete and fiber. Thus, fiber sticks to the concrete, and flexural strength is increased. Once the main crack zone becomes much stronger, the new crack may appear in a new place at the second trial of testing (testing after epoxy injection), and then a new weakest point appears. Polypropylene fibers tend to increase permeability, but to a much lesser extent compared to steel fiber. However, for the reference mix, the increase in flexural strength was slight, and this may be because the high accurate manual repairing of the beam, which was split completely, helped in that slight improvement of strength.

**Figure 13:**
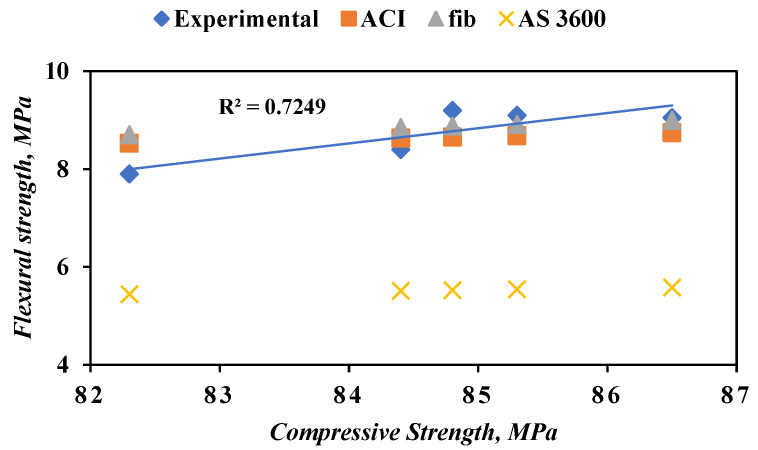
Relationship between flexural strength and compressive strength of FRSCC.

The results of the compressive and flexural strengths of FRSCC test results are correlated in Figure 13. The flexural strength of concrete can be predicted from compressive strength results using the empirical relations of suggested standards: ACI 363 [49], CEB-fib [50], and AS 3600 [51]. Among all the relations used to predict the flexural strength of concrete, ACI 363 [49] showed the closest values to the experimental results. It is worth mentioning that ACI 363 [49] is intended to be used for high-strength concrete, which is the case for the mixes used in this study. The correlation between compressive and flexural strengths is considered to be adequate (R^2^ = 0.70). Since the results of flexural strength before using the epoxy meet the predicted values proposed in the aforementioned standards, the flexural strength results after using the epoxy will do, considering the compressive strength is the same.

(1) $$f_{r}=0.94\sqrt{f_{c}}$$

(2) $$f_{r}=0.46{\left(f_{c}\right)}^{2/3}$$

(3) $$f_{r}=0.6\sqrt{f_{c}}$$

where, *f_r_* = flexural strength (MPa), *f_c_* = cylindrical compressive strength (MPa).

## 4. Conclusions

This paper is a part of a comprehensive research project undertaken to evaluate the effects of the addition of steel and polypropylene fibers on the fresh and mechanical properties of FRSCC proportioned with high strength.

The research also conducted the epoxy injection technique to strengthen the FRSCC after cracking. It was carried out on ruptured concrete specimens to assess the efficiency of the epoxy resin adhesive injection retrofitting technique for strength and stiffness. The simultaneous application of epoxy injection in FRSCC for repairing damaged concrete beams was shown to be highly effective.

According to the experimental results, the incorporation of steel or polypropylene fibers led to reduced responses in the workability of FRSCC. Such phenomena become aggravated when mixtures are proportioned with higher contents of fibers.

The impact of polypropylene or steel fibers on the compressive strength results was marginal. The positive impact of fibers on splitting tensile strength was noticeable, given that the fiber bridging action that limits crack propagation.

Steel fibers with high tensile properties could significantly improve the flexural strength and shear capacity of FRSCC, owing to the effect of fibers on the postcracking response and shear stress transfer across the cracks. Repairing FRSCC with epoxy enhances its flexural strength, unlike the normal concrete produced without fiber. The porous transition zone between the fiber and cement matrix helps to improve the strength after repair, as presented by the SEM images. Finally, the experimental results of flexural strengths were found to be predictable by several standards.

## Figures and Tables

**Table 1:** Chemical compositions and physical properties of cement.

Measured Property	CEM I
Density (g/cm^3^)	3.02
Specific surface area (cm^2^/g)	3326
Loss on ignition	3.0
SiO_2_	19.33
CaO	63.43
MgO	1.45
Fe_2_O_3_	3.42
Al_2_O_3_	4.67
SO_3_	2.6
Chloride content	0.04
Free CaO	0.71
K_2_O	0.78
Na_2_O	0.33

**Table 2:** The main properties of the used Steel and Synthetic (polypropylene) fibers.

Type	Steel Fiber 3D 65/60 BG Dramix^®^	Synthetic Fiber Macro Fiber Concrix
Material	Steel	Polyolefin
Density (g/cm^2^)	7.85	0.91
Length (mm)	60 mm	50 mm
Diameter (µm)	900 µm	500 µm
Colour	gray	yellow
Number of fibers by kg	3200	150,000
Tensile strength (MPa)	1160	590
Modulus of elasticity (GPa)	>210	>11

**Table 3:** Mixtures’ composition.

Mix	Fiber Type	Fiber Volume (%)	Cement (kg/m^3^)	Fine Aggregates (kg/m^3^)	Coarse Aggregates (kg/m^3^)	Admixture (kg/m^3^)	Water (kg/m^3^)
Control	-	0	500	783	939	1.5	175
S–0.25%	Steel	0.25	500	780	935	1.5	175
S–0.45%	Steel	0.45	500	778	933	1.5	175
P–0.25%	Polypropene	0.25	500	781	936	1.5	175
P–0.45%	Polypropene	0.45	500	779	934	1.5	175

**Table 4:** Formulas for calculating the mechanical strengths.

Test	Formula	Notations
**Compressive** **strength**	$\frac{P}{A_{C}}$	*P* = load at failure (KN) *A_C_* = compression area (mm^2^) *L_T_* = length of the cylinder (mm) *D* = diameter of the cylinder(mm) *b* = width of the prism (mm) *d* = depth of the prism (mm) *L_F_* = length between supports of the prism (mm) *A_S_* = shear area (mm^2^)
**Splitting tensile** **strength**	$\frac{2P}{\pi DL_{T}}$	
**Flexural** **strength**	$\frac{3PL_{F}}{2bd^{2}}$	
**Shear** **strength**	$\frac{P}{A_{S}}$	

## Data Availability

Not applicable.
